# Transplanted Neural Progenitor Cells from Distinct Sources Migrate Differentially in an Organotypic Model of Brain Injury

**DOI:** 10.3389/fneur.2015.00212

**Published:** 2015-10-07

**Authors:** Kapinga P. Ngalula, Nathan Cramer, Michael J. Schell, Sharon L. Juliano

**Affiliations:** ^1^Department of Anatomy, Physiology and Genetics, Uniformed Services University of Health Sciences, Bethesda, MD, USA; ^2^Department of Pharmacology, Uniformed Services University of Health Sciences, Bethesda, MD, USA

**Keywords:** neuronal migration, cerebral cortex, rat, development, interneuron

## Abstract

Brain injury is a major cause of long-term disability. The possibility exists for exogenously derived neural progenitor cells to repair damage resulting from brain injury, although more information is needed to successfully implement this promising therapy. To test the ability of neural progenitor cells (NPCs) obtained from rats to repair damaged neocortex, we transplanted neural progenitor cell suspensions into normal and injured slice cultures of the neocortex acquired from rats on postnatal day 0–3. Donor cells from E16 embryos were obtained from either the neocortex, including the ventricular zone (VZ) for excitatory cells, ganglionic eminence (GE) for inhibitory cells or a mixed population of the two. Cells were injected into the ventricular/subventricular zone (VZ/SVZ) or directly into the wounded region. Transplanted cells migrated throughout the cortical plate with GE and mixed population donor cells predominately targeting the upper cortical layers, while neocortically derived NPCs from the VZ/SVZ migrated less extensively. In the injured neocortex, transplanted cells moved predominantly into the wounded area. NPCs derived from the GE tended to be immunoreactive for GABAergic markers while those derived from the neocortex were more strongly immunoreactive for other neuronal markers such as MAP2, TUJ1, or Milli-Mark. Cells transplanted *in vitro* acquired the electrophysiological characteristics of neurons, including action potential generation and reception of spontaneous synaptic activity. This suggests that transplanted cells differentiate into neurons capable of functionally integrating with the host tissue. Together, our data suggest that transplantation of neural progenitor cells holds great potential as an emerging therapeutic intervention for restoring function lost to brain damage.

## Introduction

The cerebral cortex contains two predominant types of neurons, pyramidal–excitatory neurons (~70–85%) and inhibitory interneurons (~15–30%) (1, 2). Pyramidal neurons send axons to other areas of the cortex and distant parts of the CNS, while interneurons modulate local circuits (3, 4). Proper neocortical function requires the appropriate balance between excitatory and inhibitory neurons. An imbalance may result after brain damage such as traumatic brain injury (TBI), stroke, hypoxia, or ischemia. An excitatory/inhibitory imbalance can lead to functional deficits and various disorders including cognitive and/or motor problems (5–7), sleep disorders (6, 8, 9), or epilepsy (10–13). Injuries to the brain can damage both neuronal types, but it may be possible to replace lost or damaged cells and restore physiological balance (11, 12, 14). For the replacement to be effective, the transplanted cells should reach and remain in the target site; once there, cells must differentiate and integrate into the endogenous circuitry to restore lost function (15–17). Several studies demonstrate that neural progenitor cells (NPCs) in the ventricular/subventricular zone (VZ/SVZ) proliferate and migrate to the site of damage (18–21). Transplantation of exogenous cells in the damaged region has also been explored. GABA-expressing interneurons, or neuronal and glial precursors transplanted directly into the lesion cavity-induced and -improved sensorimotor function (22, 23). NPCs transplanted in the striatum also promote long-term functional recovery after TBI (24). Neocortical SVZ or embryonic neocortical cells transplanted in the lesion cavity remain close to the injection site but also differentiate into neurons that extend axons (15, 25). Several animal models of brain injury tested the ability of transplanted cells to repair the site of insult and suggest that cultured cells transplanted days after the injury differentiate, induce, and promote functional recovery (26–29).

To further clarify the ability of transplanted NPCs to integrate into host cortex and to investigate the cells most optimal to repopulate damaged neocortex, we used an organotypic culture model of brain injury. We compared two sources of NPCs (GE and neocortex) from embryonic rat brain for their ability to repair a model of damaged cortex. Our results show that both cell types have the capacity to differentiate into functional neurons and integrate into the host neuronal circuitry.

## Materials and Methods

We used Sprague Dawley rats (Charles River, Wilmington, MA, USA) at either embryonic day 16 (E16) or postnatal day 0–3 (P0–P3). All animal experiments were approved by the USUHS Institutional Animal Care and Use Committee (IACUC).

### Organotypic cultures and brain injury

P0–P3 rats were anesthetized with 50 mg/kg of euthasol. When animals were unresponsive to painful stimuli, the skin and skull were incised, the brain removed, and placed in oxygenated ice-cold artificial cerebrospinal fluid (aCSF) containing H_2_O; and in mM; 124 NaCl; 26 NaHCO_3_; 10 glucose; 1.2 NaH_2_PO; 3.2 KCl; 1.2 MgSO_4_; 2.4 CaCl_2_. Coronal slices (500 μm) were cut using a manual tissue chopper (Stoelting Co., Wood Dale, IL, USA) and placed on a 0.4 μm culture plate insert (Millicell-CM 30 mm, Millipore, Billerica, MA, USA) in a 6-well plate with enough media to form a meniscus above the slice. The media consisted of MEM with Earle’s salts without l-glutamine (Invitrogen, Carlsbad, CA, USA) and supplemented with 10% normal horse serum (Invitrogen), 0.001% Gentamycin, and l-glutamine (2 mM). Slices were then separated into normal and wounded groups. To simulate an injury in the cortical organotypic cultures, we used a scalpel to cut through the thickness of the cortical plate. Rat neural progenitor cells (see below) were transplanted onto the surface of the slice 6–8 h later either in the SVZ/VZ or in the injury. The slices were maintained in the incubator (5% CO_2_, 95% O_2_, 37˚C) for 5–7 days to allow transplanted cells to migrate or settle into the slice and integrate the host tissue.

### Cell suspension preparation

Embryos were taken from timed pregnant rats on embryonic day 16 (E16), the brains were removed and placed into ice-cold aCSF under aseptic conditions. The rostral and caudal poles were removed and the meninges peeled away and the cortical plate and ganglionic eminence (GE) separately dissected and placed in oxygenated ice-cold aCSF. The GE was removed from the surrounding tissue, while the cortical wall was dissected to remove the developing cortical plate and retain the VZ, SVZ, and part of the IZ (intermediate zone). The tissues were then transferred to two separate 15-ml centrifuge tubes containing phosphate buffered saline (PBS) without calcium and magnesium (Quality Biological, Gaithersburg, MD, USA) plus 0.6% glucose (EMD, Gibbstown, NJ, USA); each tube contained either neocortical or GE tissue. The solution plus tissue was mechanically triturated with a series of 9-inch fire polished Pasteur pipettes to form a single cell suspension. Cell density was determined and cells prepared for labeling, transfection, and transplantation. Cell viability was tested using trypan blue exclusion; we also tested the viability after labeling the cells (see below). Only cell suspensions with viability of 75% or greater were used. Cells were then maintained in three different groups: the neocortex alone, the GE alone, or a mixed population of both cortical and GE cells.

### Cell suspension labeling

After cell counting, we added 5 μl of Vybrant CM-DiI (Invitrogen) to 1 million cells suspended in 1 ml of warm PBS without calcium and magnesium, with 0.6% glucose. Cells were then incubated for 15 min at 37°C and mixed every 3–5 min to distribute the dye evenly. After incubation cells were spun at 1000 rpm for 10 min and washed several times using warm PBS plus 0.6% glucose, to remove the excess dye. After washing, the cells were resuspended into a small volume of PBS to have a final concentration of 10^5^cells/μl. The use of CMDiI is justified by our previous work using similar techniques. We observed previously that this dye does not leak into the tissue upon transplantation of dead labeled cells, and we did not observe any subsequent movement of either dead cells or fluorescent particles moving away from the injection site (30).

We also used transfection to identify the transplanted cells, after quantification and viability testing. A cohort of cells was transfected with plasmid DNA expressing GFP under a CMV promoter, using a calcium phosphate method (31). To do this, a calcium–DNA precipitate was added to the cell suspension for 1.5 h. The precipitate was removed and cells washed with transfection media (Neurobasal (Invitrogen) plus in mM, 10 MgCl_2_, 10 HEPES and pen/strep at 1:100) before transplanting as described below.

### Cell transplantation

Prior to transplantation, organotypic cultures were placed in an incubator for 4–6 h. One hundred nanoliters of cell suspension (10^5^ cells/μl) were injected onto a specific region of the culture, using a controlled nanoliter injector (WPI, Sarasota, FL, USA) and a pulled micropipette of ~30 μm tip size. For cultures without an injury, cells were transplanted into the VZ/SVZ only, while in the injured cultures, cells were transplanted into the VZ/SVZ or into the wounded region. After injection, the pipette remained in place for 1–2 min to allow the cells to settle on the surface of the slice. Cultures were then returned to the incubator for 2 h to ensure adherence, before adding more media to adjust the meniscus above the slice. The cultures were maintained every 2–3 days by removing half of the media, which was replaced by the same amount of fresh media.

### Electrophysiological recordings

Only transfected cells transplanted into injured slices were used for the electrophysiological studies. After 7 days in culture, slices were transferred to a submersion style recording chamber; and perfused with room temperature, aCSF continuously bubbled with 95/5% O_2_/CO_2_. The aCSF contained (in mM): 126 NaCl, 3 KCl, 1.25 NaH_2_PO_4_, 2 MgSO_4_, 26 NaHCO_3_, 2 CaCl_2_, and 10 d-glucose. Whole cell recordings used an Axopatch 200B or Multiclamp 700B amplifier, digitized by a Digidata 1322A, and stored on a PC running pClamp version 9 software (Molecular Devices, Sunnyvale, CA, USA). The intracellular solution was composed of (in mM): 130 K-Gluconate, 15 KCl, 5 HEPES, 1 EGTA, 4 Mg–ATP, and 0.3 Na–GTP. In a subset of recordings, 0.2% Neurobiotin™ Tracer (Vector Laboratories, Burlingame, CA, USA) was included in the intracellular solution to allow *post hoc* visualization of recorded cells. Whole cell recordings took place in the vicinity of the injured region. Cells without any GFP in the injury site and away from it were recorded to serve as a control for electrophysiological characterization.

### Immunohistochemical analysis

After 5–7 days, cultures were fixed in 4% phosphate buffered paraformaldehyde overnight. Tissue was washed in 0.1M PBS pH 7.4 three times and subsequently blocked for 2 h in PBS normal goat serum with 0.1% Triton-X. The primary antibody was prepared in the blocking solution and applied in the following dilutions: anti-GFAP 1:500 (Abcam, Cambridge, MA, USA), anti-GABA 1:500, anti-TUJ1 1:100 and anti-MAP2abc 1:100 (Sigma-Aldrich, St. Louis, MO, USA), and Milli-Mark Pan Neuronal Marker 1:25 (Millipore) for 2 h at room temperature on a shaker and then left for 24–48 h at 4°C. The appropriate secondary antibody 1:500, Alexa 488 or Alexa 546 (Invitrogen) was applied for 2 h and each tissue section washed three times with PBS. Each section was incubated in a 2 μg/ml solution of bisbenzimide for 5 min to label nuclei. The sections were then mounted in Vectashield mounting medium for fluorescence (Vector Laboratories) or Mowiol 4-88 (Sigma-Aldrich) and coverslipped. To visualize cells that were injected with Neurobiotin each recorded slice was fixed with 4% buffered paraformaldehyde at 4˚C overnight in the dark. The slice was then immunoreacted with an avidin–rhodamine conjugate (Vector laboratories) and mounted with proLong gold antifade reagent with DAPI (Invitrogen).

### Quantification and statistical analysis

Numbers of cultures used are presented in Tables 1 and 2. To assess the distribution of cells migrating away from the transplant site in the VZ/SVZ, we counted cells that (1) migrated away from the injection site for at least 200 μm, and (2) were labeled with CMDiI (cell body alone or cell body with at least one process). To delineate the area of migration, bisbenzimide images were used to visualize the cortical plate and the intermediate zone. The cortical plate was subdivided in three equal subdivisions corresponding to upper, middle, and lower regions. The hemisphere of each organotypic culture was also divided into lateral, middle, and medial regions to assess the mediolateral distribution of transplanted cells. Adobe Photoshop and Image J (NIH, USA) were used to analyze the images. To compare across slices, the cell count in different regions or in different layers was expressed as the percent of the total number of migrated cells. Cells injected directly into the injury were not quantified as they remained in place without showing any migration pattern. Statistical analysis for each group used an ANOVA (two way) and the Holm–Sidak *post hoc*-test for multiple comparisons.

**Table 1:** **Total number of cells counted in each layer and region of the cortical plate after transplantation into organotypic cultures**.

Cell source	No. of slices	Total number of counted cells	Regional distribution of counted cells							
			U	M	L	CP	IZ	Medial	Middle	Lateral
GE	11	23556	8632	7219	4804	20655	2901	6522	8475	8559
cortical	11	21657	4911	5342	5555	15808	5849	5863	7546	8248
Mixed	10	20386	7430	6055	4344	17829	2557	5261	6814	8311

*The transplanted cells were identified by CMDiI label*.

**Table 2:** **Total number of transplanted cells counted in the injured and uninjured regions of cortical plate**.

Cell source	No. of slices	Total number of counted cells	Cell distribution		
			Injured area of the cortex	Uninjured area of the cortex	IZ
GE	14	20727	8574	7868	4285
Cortical	16	23034	7923	7761	7350
Mixed	16	22371	9104	8764	4503

*All transplanted cell were labeled with CMDiI*.

## Results

**Figure 1:**
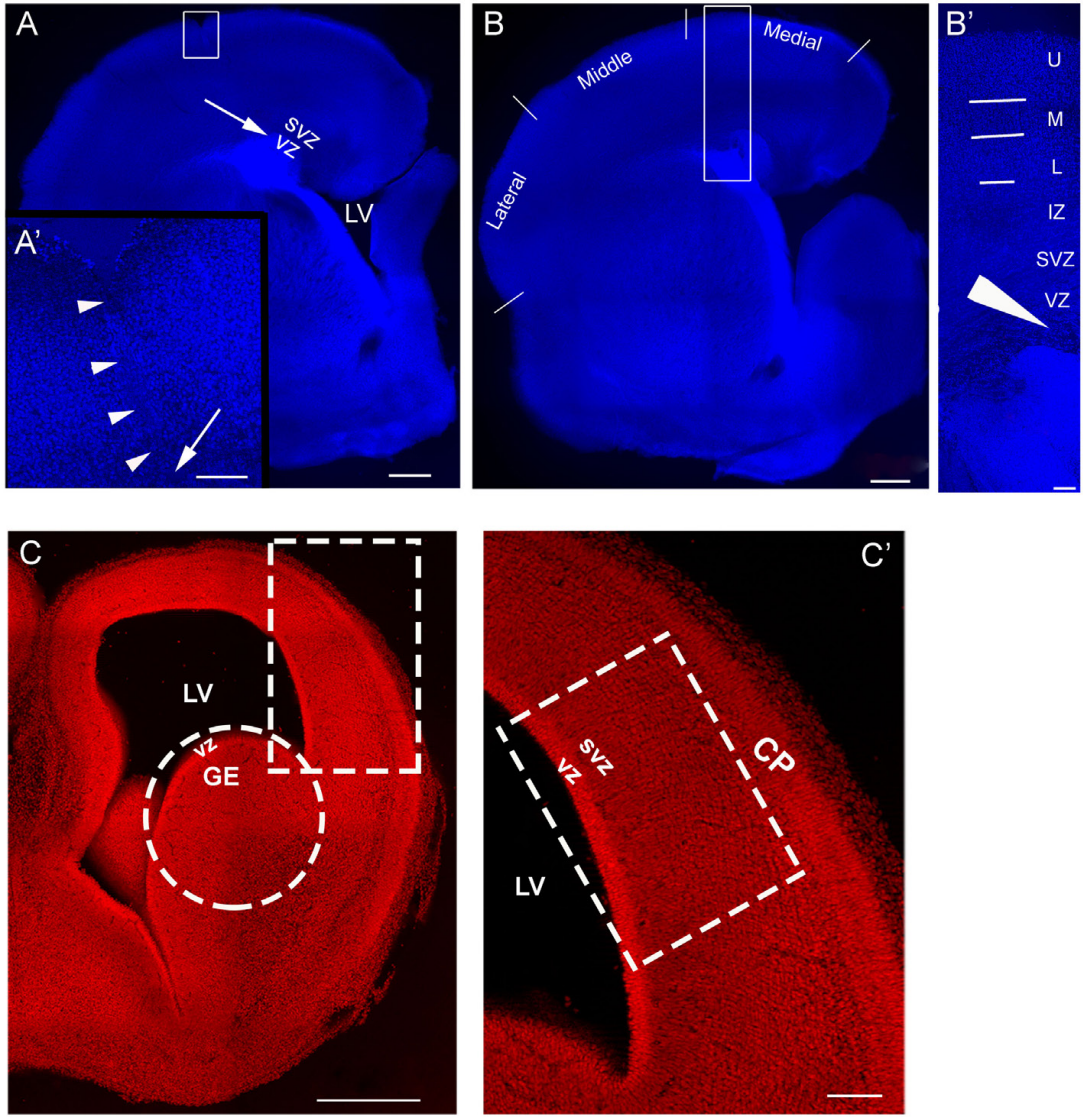
**Model of injury and transplantation**. **(A)** is an organotypic culture obtained on postnatal day 1 (P1), that also sustained an injury. The arrow is the approximate site of a transplant into the SVZ/VZ. **(A′)** shows the boxed area in **(A)** and contains a higher power view of the injury outlined by arrowheads; the arrow points to the deep end of the lesion. **(B)** is an organotypic culture obtained at P1 without injury. For analysis, the neocortex was divided into regions designated as medial, middle, and lateral. **(B′)** is a higher magnification of the boxed in region in **(B)** and delineates laminar distinctions in the cortical plate used to define the positions of the transplanted migrating cells. The arrow in **(A′)** represents the approximate site of cells injected into a lesion and the arrow head in **(B′)** represents the approximate site of cells injected into the VZ/SVZ of a slice with no lesion. **(C)** is a coronal section of E16 brain, **(C′)** is higher magnification of the boxed in region in **(C)** and represents the developing cortical wall. This is the region used for preparing the cell suspension made from the embryonic neocortex. The circle in C encloses the site used for preparing the GE cell suspension. CP: cortical plate, U: upper layer, M: middle layer, L: lower layer, IZ: intermediate zone, SVZ, subventricular zone; VZ, ventricular zone; LV, lateral ventricle. Scale bar = 500 μm **(A–C)**, 100 μm **(A′–C′)**.

An example of an organotypic culture containing a lesion can be seen in Figure 1A. For analysis, the slice was divided into medial, middle, and lateral regions (Figure 1B). The immature cortical plate was divided into upper, middle, and lower layers (Figure 1B′). The approximate sites of transplantation are indicated by an arrow (Figures 1A,A′,B′). Cells obtained acutely from E16 rat embryos (Figures 1C,C′) were transplanted into the VZ/SVZ of a neocortical slice in control and injured cultures as indicated by the arrows in Figures 1A–B′.

### Migration pattern of cells transplanted into the VZ/SVZ of control slices

**Figure 2:**
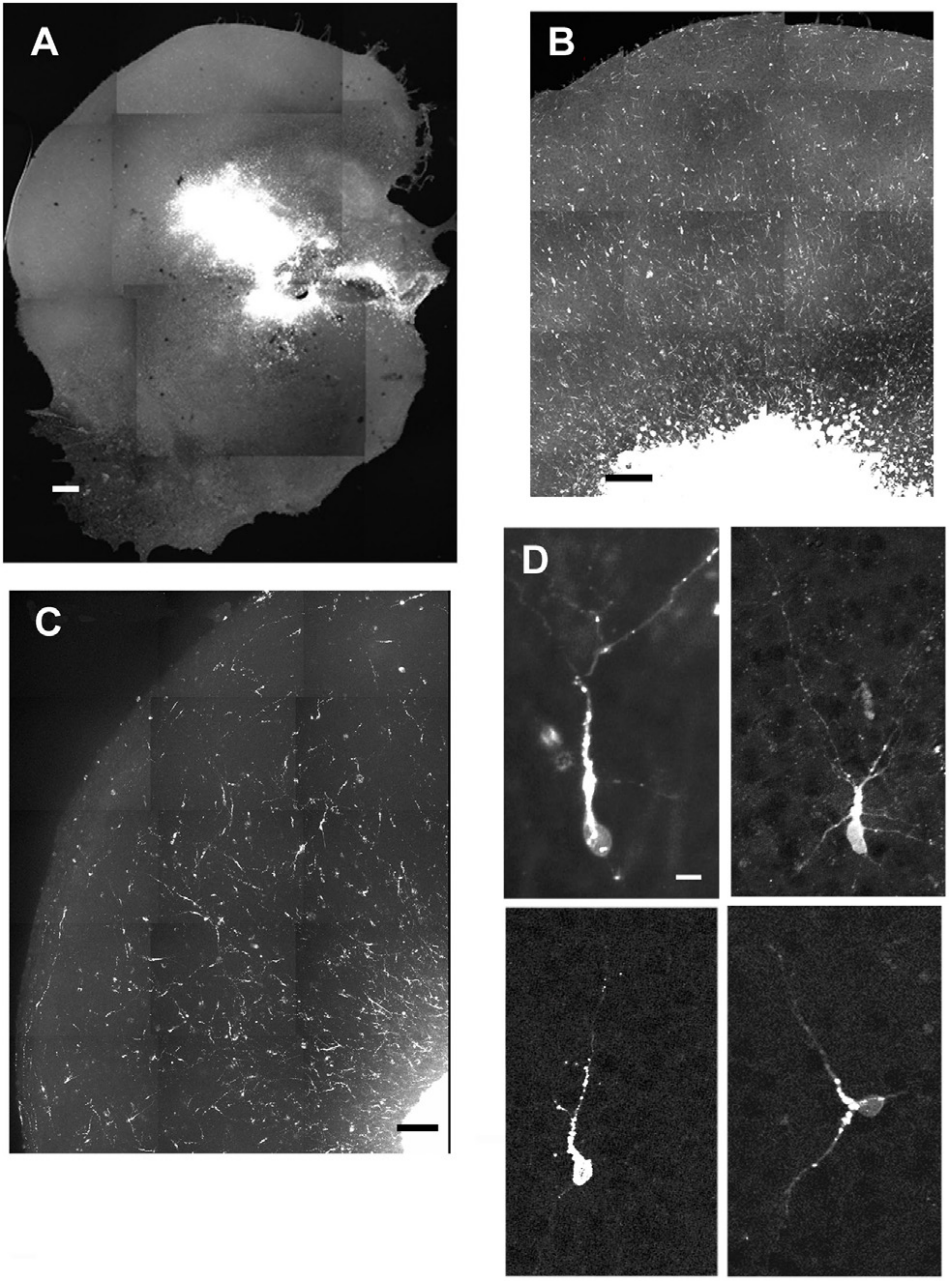
**Examples of cells migrating after transplantation into normal cultures**. **(A–C)** are examples of different slices that received injections of acute cell suspensions obtained from the GE **(A,B)** or the neocortex **(C)**. All populations of transplanted cells (ganglionic eminence, mixed, or neocortical) showed similar distributions throughout the cortex. **(D)** shows morphologies acquired by the transplanted cells. Scale bar = 500 μm **(A)**, 100 μm **(B–C)**, 10 μm **(D)**.

**Figure 3:**
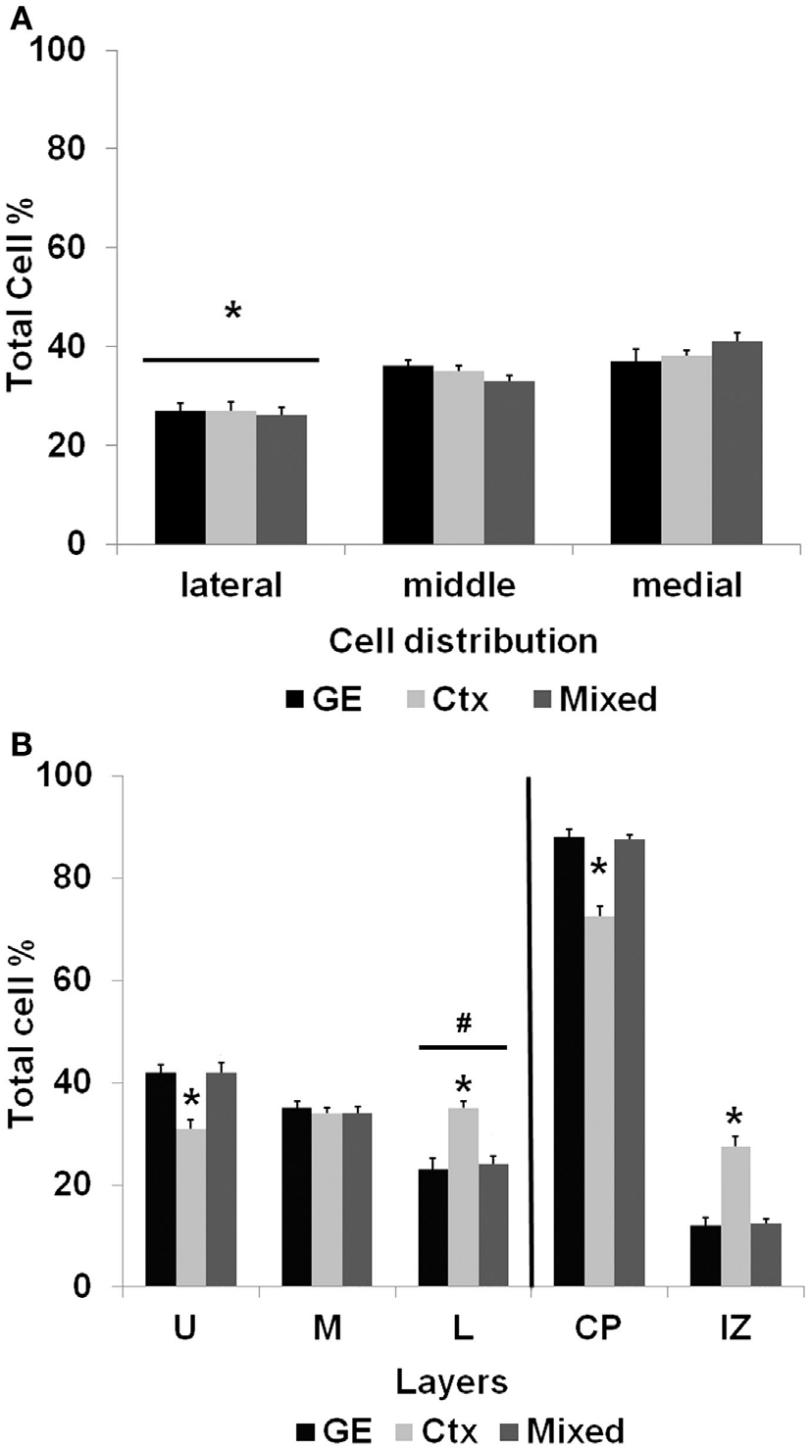
**The distribution of transplanted cells in normal cultures**. **(A)** shows the mediolateral distribution of cells transplanted into normal cortex after 7 days in culture. Significantly fewer cells reached lateral portions of the cortical slice compared with transplanted cells migrating into the middle and medial regions of the slice. **(B)** illustrates the distribution of cells that migrated away from the injection site. The bars to the right of the vertical line show that of the cells migrating away from the injection site, a greater percentage reached the cortical plate (CP) than those remaining in the intermediate zone (IZ). Significantly fewer cells obtained from the neocortex alone, however, reached the cortical plate compared with cells obtained from the GE or the mixed population (*p < 0.01). The bars to the left of the vertical line show the distribution of cells that reached the cortical plate and moved into the upper (U), middle (M), or lower (L) cortical layers. Of the cells that reach the cortical plate, fewer cells resided in the lower layers of the cortical plate (^#^*p* < 0.05). Compared with the GE and mixed populations, a significantly smaller percentage of neocortically derived cells reached the upper layers (*p < 0.001) and a greater percentage of cells remained in the lower layers (*p < 0.001). See Table 1 for the numbers of cells in each group. (Two-way ANOVA followed by the Holm–Sidak pairwise comparison, error bars = SEM).

After the slices were placed in culture, they received transplants of acutely derived cell suspensions obtained from the neocortex alone, the GE alone, or a mixed population of neocortex and GE. Based on the count of viable transplanted cells and the number of transplanted cells, we approximated the number of cells that migrated away from the injection site. About 20% of the total amount of transplanted cells migrated away from an injection suggesting a similar viability percentage. In normal uninjured slices, each cell population moved into the cortical plate and migrated extensively into medial, middle, and lateral parts of the slices (Figures 2A–C; also see Table 1). After reaching their destination, the transplanted cells acquired different morphologies and displayed multiple processes and neuron-like morphologies (Figure 2D). All of the transplanted cell types (neocortical, GE, and mixed) distributed similarly in the different regions of the host slice. When we quantified the medial to lateral distribution of transplanted cells, we observed that cells of all groups migrated in greater numbers toward the medial portions of the host cortical slice, whereas fewer cells moved into lateral regions (Figure 3A; Table 1). More cells derived from any source moved into the middle and medial regions of the CP (Figure 3A). Most of the migrating cells moved through the intermediate zone into the cortical plate (Figure 3B). More cells derived from the GE and mixed cell population, however, reached the cortical plate than those derived from the neocortex, leaving a greater percentage of neocortically derived cells in the intermediate zone (Figure 3B; Table 1). In the CP, GE cells are in great number located into the upper layer while neocortical cells are preferentially in the middle and lower layers. These results suggest that: (i) all populations of transplanted cells (GE-derived, neocortical-derived, and mixed) migrate preferentially toward the middle and medial CP, (ii) GE-derived cells migrate more efficiently than cortical cells, and (iii) the migration of ­neocortically derived cells is improved when transplanted together with GE-derived cells.

### Migration pattern of cells transplanted into the VZ/SVZ of injured cultures

**Figure 4:**
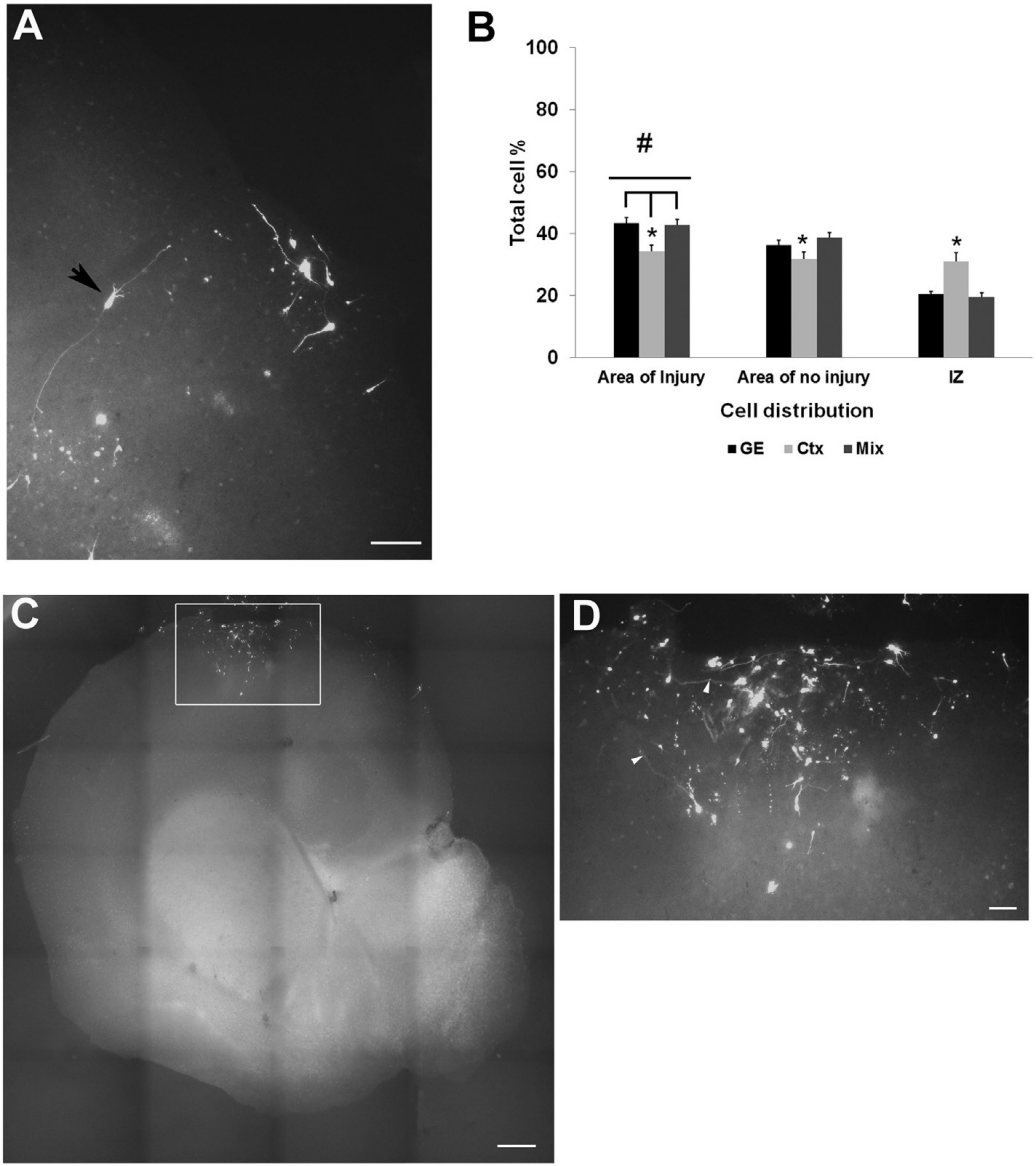
**Transplanted cell distribution in injured cultures**. **(A)** shows examples of transfected GFP cell entering or in the injured area. An arrowhead points to a cell seeming to extend processes within the injured area. **(B)** is a graph of the distribution of CMDiI labeled cells transplanted into the SVZ of an injured culture that migrated into both injured and the non-injured regions of host slice. Significantly more cells migrated into the injured region (^#^*p* < 0.001) compared to the areas of no injury. Fewer neocortical cells reached the injured area compared to GE and mixed (*p < 0.05) cell populations. More of the neocortically derived cells remained in the IZ compared with the other two populations (*p < 0.05). (Two-way ANOVA followed by a *post hoc* pairwise comparison, Holm–Sidak, error bars = SEM). **(C,D)** Cells transplanted in the injury remained in place and did not show any migration. **(D)** is a higher powered image of the boxed in region in B. [Scale bar **(A)** = 100 μm; **(C)** = 500 μm; **(D)** = 20 μm]. IZ, intermediate zone; GE, cells derived from the ganglionic eminence; Ctx, cells derived from the embryonic neocortex; Mix, cells derived from a mixed population of GE and neocortically derived cells.

To study the effect of an injury on the ability of NPCs to migrate into and populate host cortex, mechanical damage was made through the thickness of the neocortex in organotypic cultures; cells were transplanted into the VZ/SVZ as shown in Figure 1A. Transplants of all cell types into the VZ/SVZ demonstrated a targeted migration toward the lesioned zone (Figure 4A). Migrating cells also populated the area of the non-lesioned neocortex, but to a lesser extent. Cells derived from the neocortex were less efficient in migration compared to GE-derived and the mixed cell population in that more cells tended to remain in the IZ and distribute throughout the host slice (Table 2; Figure 4B). When cells were transplanted directly into the lesioned zone, they remained in that region, showing little signs of migration or moving away from the lesion site (Figures 4C,D). This was true for all transplanted cell types (derived from the neocortex alone, from the GE alone, or the mixed cell population), which all remained in the lesion.

### Phenotype of transplanted cells

**Figure 5:**
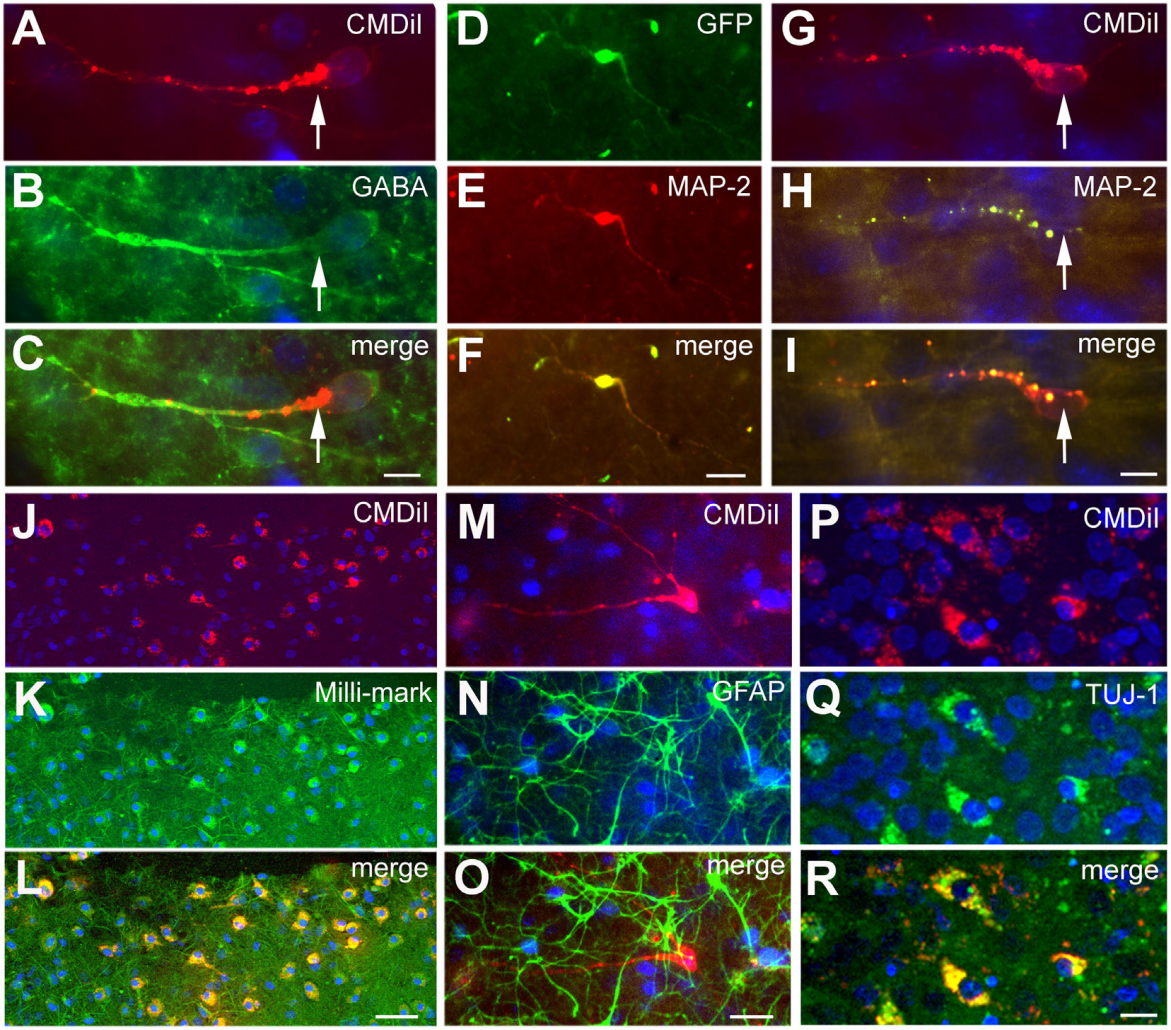
**Phenotype of transplanted cells**. The transplanted cells acquired different phenotypes as shown by immunoreactivity against different neuronal and glial markers. The cells shown in **(A–C)** derive from the GE and are GABA+. Cells shown in **(D–I)** derive from the neocortex, and the cells shown in **(J–L)** are a mixed population of transplanted cells; the cells in **(D–L)** and **(P–R)** are immunoreactive for neuronal markers, MAP2 in **(D–I)**, Milli-Mark in **(J–L)**, and TUJ in **(P–R)**. The cells in **(M–O)** show GFAP+ cells (green) in the host slice and a transplanted cell from the GE labeled with CMDiI (red), which is not GFAP immunoreactive. **(D–F)** is a transfected GFP+ (green) cell and **(G–I)** are CMDiI+ (red). **(J–K)** are CMDiI+ (red) and Milli-Mark+ (green). Scale bar = [**(A–C,G–I,P–R)** 10 μm] and [**(D–F,J–O)** 50 μm].

**Figure 6:**
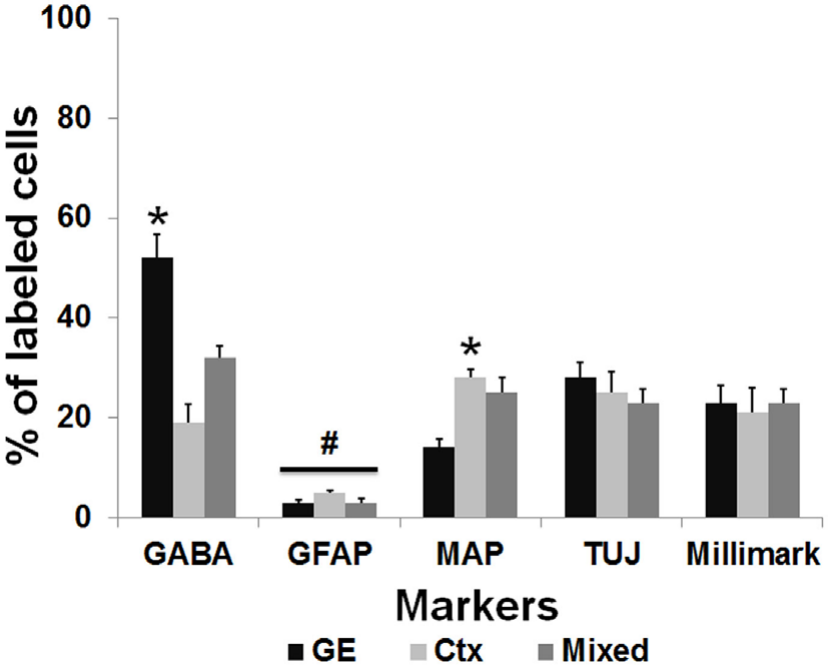
**Transplanted cells immunoreactive for different markers**. This graph represents the percentage of the total number of transplanted cells labeled with CMDiI that were counted for each marker, which were also double labeled for a specific antibody. The total number of counted cells for each marker can be seen in Table 2. In general, similar numbers of transplanted cells from derived from each source (GE, Ctx, Mixed) were immunoreactive for the neuronal markers, Tuj1 (TUJ) and Milli-Mark. GE-derived cells were more likely to differentiate into cells immunoreactive for GABA, while neocortically derived cells are more immunoreactive for MAP2 (*p < 0.01). Very few transplanted cells from any source are immunoreactive for GFAP (^#^*p* > 0.05). We used a two-way ANOVA followed by pairwise comparisons with the Holm–Sidak test, error bars = SEM.

To further characterize the phenotype of the transplanted cells, the organotypic culture slices were fixed at day 5 or 7 post transplantation and immunoreacted for neuronal and glial markers. The distinct morphologies of the transplanted cells, as shown in Figure 2D, suggests that they were differentiating into well-defined neural cell types. To more completely assign a phenotype to the transplanted cells, we used a battery of markers to further characterize their identity. In addition, the NPCs transplanted from different embryonic sources might differentiate into distinct neuronal types. We used several neural markers: GABA, MAP2, β-tubulin III (TUJ-1), Milli-Mark, and GFAP (Figure 5). The labeled transplanted cells showed immunoreactivity for multiple markers, suggesting they differentiated into a variety of cell types. Figure 5 demonstrates transplanted cells labeled with CMDiI or GFP and immunoreactive for neuronal (Figures 5A–L,P–R) or glial markers (Figures 5M–O). Figures 5A–C shows a cell labeled with CMDiI displaying a migratory morphology immunoreactive against GABA. The images in Figures 5D–L illustrate examples of labeled transplanted cells reactive for other neuronal markers, including MAP2 (D–F and G–I), Milli-Mark (J–L) and TUJ (P–R). Figures 5M–O shows an example of a CMDiI labeled cell surrounded by GFAP immunoreactive cells, demonstrating that many GFAP+ cells occurred in each organotypic slice, but very few of the transplanted cells were GFAP+. Figure 6 shows the percent of transplanted cells immunoreactive for all markers across experiments and also illustrates the fraction of cells from each source that displayed immunoreactivity out of the total number of cells that were transplanted. Transplants derived from the GE were more likely to differentiate into GABAergic cells than neocortically derived cells. All populations of cells demonstrated very low reactivity for GFAP, suggesting that very few of the transplanted cells differentiate into astrocytes. The numbers of cells counted and used to produce Figure 6 can be seen in Table 3.

**Table 3:** **Total number of transplanted cells double-labeled with CMDiI and another marker for analysis of distinctions in immunoreactivity of cells obtained from different sources**.

Cell type	No. of slices	Total number of double labeled cells				
		GABA	GFAP	MAP	TUJ	MilliMark
GE	41	901	64	330	988	699
Cortical	37	255	86	611	672	543
Mixed	42	894	65	720	833	648

### Transplant integration and functional analysis

**Figure 7:**
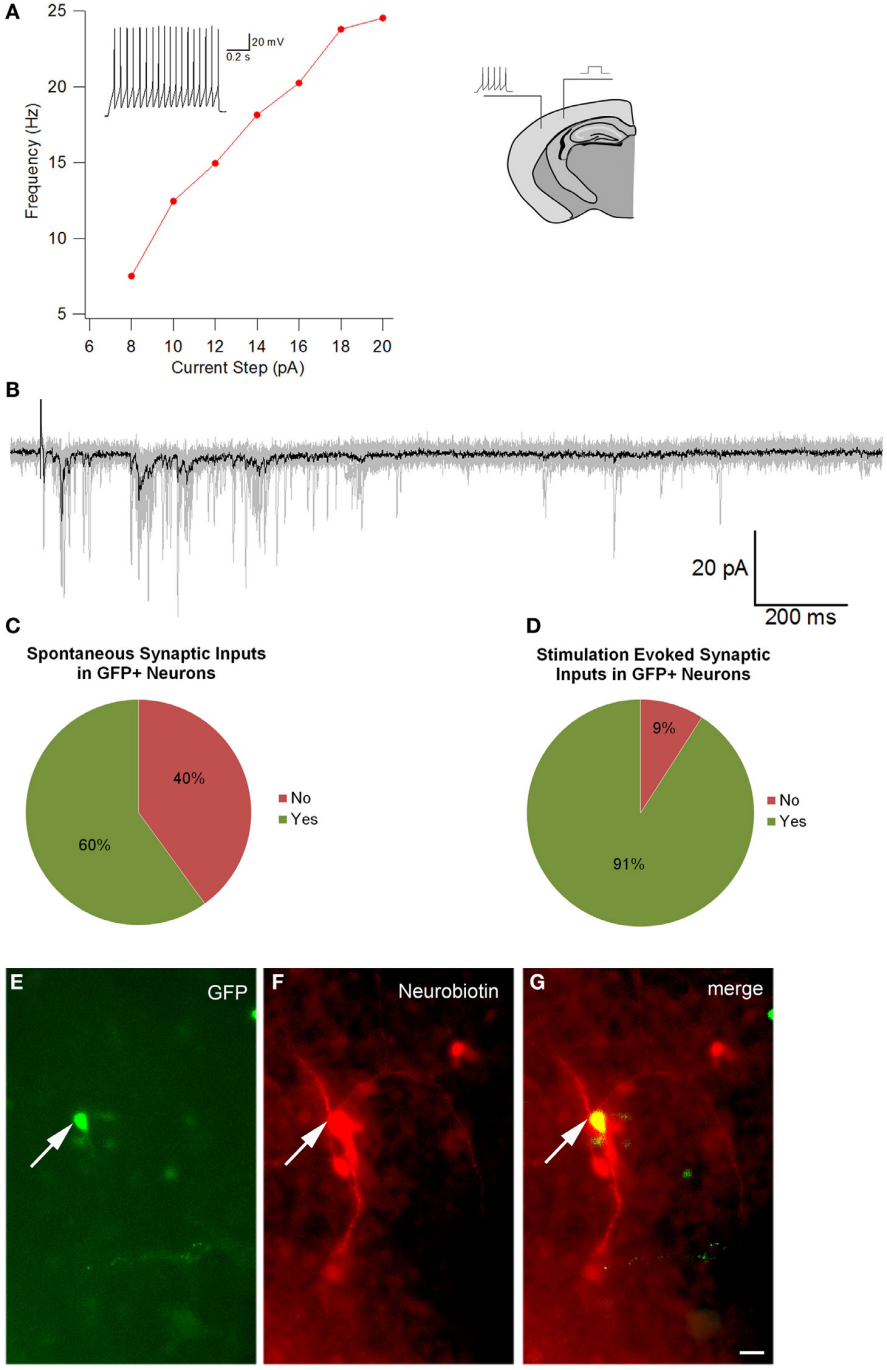
**Electrophysiological recordings**. **(A)** is the Current–frequency relationship of a GFP+ transplanted neuron. The recorded cell showed a linear increase in firing frequency as a function of depolarizing current. The inset in the graph shows the response of the cell to a 14pA depolarization. Note the lack of spike frequency adaptation, a characteristic of fast spiking interneurons. **(B)** Intracortical stimulation evoked postsynaptic currents in the same neuron. The black trace is the average of 10 individual sweeps (each sweep overlaid in gray). The prolonged barrage of PSPs impinging upon the cell suggests that the transplanted neuron had become integrated into the host circuitry. The image of the brain drawing above shows a schematic representation of the position of a stimulating electrode and site of recording of a labeled cell. **(C)** Percentage of GFP cells that demonstrated spontaneous synaptic inputs (*n* = 15), and **(D)** percentage of GFP cells that showed synaptic activity after electrical stimulation (*n* = 11). **(E–G)** An Avidin–Rhodamine reaction cell recording, **(E)** shows GFP, **(F)** shows the Neurobiotin reaction and **(G)** merge of the two. Scale bar: 20 μm.

To optimize our electrophysiological analysis, whole cell recordings were taken from GFP positive transplanted as well as concomitant host cells, which were not labeled, after up to 7 days in culture. These experiments revealed that transplanted cells had significantly more depolarized resting potentials than host cells (Table 4, −30 ± 3 mV, *n* = 11 versus −52 ± 8 mV, *n* = 4; *p* = 0.048) with 5 out of 16 transplant and 1 of 5 host cells firing action potentials spontaneously at rest (Figures 7C,D). Transplanted cells tended to have lower cell capacitances (18 ± 3 pF, *n* = 16 versus 30 ± 6 pF, *n* = 5; *p* = 0.11) and resistances (1580 ± 340 MΩ versus 620 ± 70 MΩ; *p* = 0.18) (Table 4). Most of the transplanted cells acquired electrophysiological characteristics of neurons with properties that reflected their comparatively immature age. Spontaneous synaptic inputs were present in 60% of the transplanted neurons examined (9 of 15) (Figure 7C) and, in 11 cells tested, 10 (91%) showed synaptic activity evoked by electrical stimulation of the cortex outside the transplant location (Figure 7D). Figure 7A shows an example of a cell stimulated by activating a cortical region some distance away; increasing the current also increased the frequency of firing. Figure 7B shows a similar cell demonstrating synaptic inputs after being stimulated from some distance away in the neocortex (e.g., inset in Figure 7A). After recordings, cells were immunoreacted with an avidin conjugate demonstrating their extended processes as seen in Figures 7E–G. These observations suggest that transplanted cells acquire neuronal phenotypes and integrate into the host tissue, highlighting their therapeutic potential.

**Table 4:** **Passive cell membrane properties of recorded neurons**.

	Resting potential		Cell capacitance (pF)		Membrane resistance (MΩ)	
	GFP	Non-GFP	GFP	Non-GFP	GFP	Non-GFP
Average	−30	−52	18	30	1600	620
SEM	3	8	2	6	340	75
*n*	11	3	16	4	16	4

## Discussion

Organotypic slices remain viable for relatively long periods of time and maintain the structure and anatomical relationships within the neocortex. The rate of cell death is low and cell proliferation and migration under appropriate conditions correspond to migration occurring *in vivo* (32–34). Organotypic cultures also offer the advantage of preserving the synaptic and anatomical organization of *in vivo* neuronal circuitry (35). In this study, we used an organotypic slice paradigm to evaluate the ability of NPCs obtained from different sources to repair the injured cortex. After brain damage, the loss of both excitatory and inhibitory neurons can lead to an imbalance of cortical responses. To repair the injured neocortex with select combinations of excitatory and inhibitory cells, we transplanted NPCs from different sources and phenotypes into separate host sites to determine their ability to repopulate and integrate into the host cortex.

### In intact cortex, GE-derived neurons migrate more efficiently than cortically derived neurons

The neocortex contains cells that originate from the GE as well as cells generated in the neocortical VZ/SVZ. Cells originating from the GE migrate tangentially to the neocortical targets, thus traveling a longer distance than cells originating from the neocortical VZ/SVZ, which travel a shorter radial distance (4). GE-derived cells differentiate into GABAergic interneurons, which are inhibitory, while neocortically derived cells tend to differentiate into projection neurons, which are excitatory (3, 4). We used both cell types here to evaluate the ability of each cell phenotype to migrate into and populate the neocortex.

In the uninjured cultures, all donor types of NPCs transplanted into the VZ/SVZ migrated toward, and reached the cortical plate. This suggests that the transplanted cells were responding to cues that regulate cell migration and positioning. Known cues include reelin (36–38), netrins (39), and the chemokine SDF-1 (40). The GE-derived cells moved into the upper layers of the cortical plate, while fewer of the neocortically derived NPCs reach this destination and tended instead to accumulate in the lower cortical plate. Migrating interneurons receive cues from cortical plate projection neurons, which could easily provide information to our transplanted interneurons (41). The projection neurons may not provide the same cues to themselves, i.e., the neocortically derived neurons, which could explain their failure to migrate into the upper cortical layers. In addition, interneurons follow different routes of migration toward their final resting place in the cortical plate (41, 42), which may contribute to their mobility when transplanted into organotypic cultures. Some of our transplanted GE-derived cells may have been heading for the marginal zone (future layer 1), which is one of their normal migratory routes, resulting in more cells in the upper layers. GE-derived neurons also migrate more quickly than those originating from the neocortex, which would facilitate their ability to reach a more distant target (3, 4, 43).

Neocortically derived neurons migrate radially and rely on the physical support of the radial glial cells to attain their target within the cortical plate (44–46). At P0–P3, although radial glia begin to disappear or differentiate into astrocytes, much of the radial glial scaffold remains in place (47, 48). Since neocortical neurons generated on E16 in rat embryos normally reside predominantly in the lower layers (4, 44–46, 49), the transplanted cells derived from the neocortex may migrate into their normal lower layer target site and remain there. The GE-derived cells, however, may typically be more mobile and have the capability to move into multiple cortical sites. When the neocortically derived cells are mixed with the GE derived cells, the mixed population showed a more extensive migration. Although we did not specifically investigate why this happens, it may be that GE-derived cells provide signals to cortically derived neurons. One of the substances that customarily provide migratory cues is a gradient of GABA itself (50–52). As the GE-derived cells are GABAergic, they might provide a source of GABA, facilitating the migration of cortically derived neurons toward their target. We do not know why the transplanted NPCs predominantly maintained their phenotype of origin. Clearly, the total environment must play a role in determining their ultimate destiny since the entire population of transplanted cells from each group did not become either GABA immunoreactive (presumptive inhibitory) or MAP2 immunoreactive (presumptive excitatory). We also did not test other markers that may have revealed subtle distinctions within the population of transplanted cells. Although the NPCs appear to contain the necessary programing to generate specific neuronal subtypes (53), their ultimate destination and degree of integration is also influenced by exogenous environmental cues.

### NPCs are attracted toward the site of injury

Several studies report that transplanted cells migrate into, or remain in, an area of lesion (54–58). To replace cell populations missing after brain damage, transplanted cells have been inserted into multiple sites including the lateral ventricle (54), the VZ/SVZ (24), the injured region (16, 22, 54, 55, 59), or near the vicinity of the injury (15, 23). All of these sites have varying levels of success in terms of cell survivability and integration into the host tissue (17, 30, 54, 60). We tested two sites of transplantation in the current study: the VZ/SVZ and the injury site. We found that NPCs transplanted into the VZ/SVZ of injured organotypic cultures migrate extensively toward the injury. If the transplanted migrating cells rely on environmental cues to reach their final destination, these findings suggest that the lesioned zone contains greater attractive cues than those in the surrounding intact neocortex. A number of other studies report that transplanted NPCs show targeted migration to the area of brain damage and become integrated into the host (54, 55, 58, 61), Most of these studies transplant cells into brains injured by stroke or examined a more targeted lesion, such as the substantia nigra in Parkinson’s Disease, but it appears that analogous patterns operate in our brain injury model. We also observed that NPCs transplanted directly into the injury site remain in place. Presumably, these cells stay because they respond to attractive signals within the injury site. The most likely attractive cues are chemokines and cytokines (62, 63). Although an injury can be considered as a hostile environment, released inflammatory molecules, such as stromal cell-derived factor α1 (SDF1), attract neural stem cells causing the injury environment to retain the transplanted cells (63). Chemokine expression is upregulated in regions of injury (64). In experiments using mice with chemokine receptors or monocyte chemoattractant protein knocked out, targeted migration of transplanted cells after induced inflammation in the hippocampus was diminished (62). A similar finding was observed after middle cerebral artery occlusion in mice with either the receptor or monocyte chemoattractant protein eliminated; infusion of monocyte chemoattractant protein also induced migration of cells to the site of infusion (64). Although we did not specifically assess the presence of chemokines or cytokines in our experiments, it is likely that they were present and attracted or retained the transplanted NPCs.

### Transplanted cells differentiate into neurons and integrate into host neocortex

Transplanted NPCs tend to acquire a neuronal phenotype when placed into an injured cortex as reported by others, while very few cells tend to differentiate into astrocytes (28, 58, 65, 66). In the current experiments, this was true for all transplanted cells whether they were headed for the injured region or not. The cells derived from the GE were GABA immunoreactive in significantly higher percentages than the neocortically derived or mixed population, suggesting that the transplanted NPCs retained their phenotype after placement in a novel environment and migration into the host cortical plate. Similarly, the cells obtained from the neocortical VZ were MAP2 immunoreactive in significantly greater numbers than GABAergic, suggesting that this population tended to differentiate into projection neurons. In support of this idea, Götz et al. reported that neocortically derived cells differentiated more into glutamatergic (excitatory) neurons than GABAergic (inhibitory) neurons (67). Approximately, 20–30% of transplanted cells were positive for antibodies against neuronal markers, while very few grafted cells were immunopositive for GFAP. We did not test for the presence of other glial cells, including oligodendrocytes or microglia, and these could account for some of the non-immunoreactive cells (24, 61). They could also be immunoreactive for numerous specific neural markers for which we did not test. We previously demonstrated that NPCs harvested from relatively mature mouse embryos (embryonic days 14–16) and transplanted into neocortical slices are capable of expressing proteins associated with specific neocortical layers as Cux1, ER81, and RORβ (68). We are also aware that young brains are much more permissive and more likely to encourage migration and survival after transplantation (69–71). It will also be important to study transplant efficacy in adult tissue where the degree of engraftment of stem cells tends to be diminished. Overall, our findings are highly supportive of the idea that the source of NPCs is significant and that the transplanted cells retain their identity in the host cerebral cortex.

Our electrophysiological studies also strongly suggest that the transplanted cells differentiate into functional neurons. Injection of depolarizing current, as a surrogate for endogenous synaptic input, evokes action potentials in the cells and stimulation of surrounding cortical regions causes barrages of postsynaptic activity in the transplanted cells. In addition to evoked synaptic activity, we observed spontaneously occurring synaptic events, further suggesting that cells transplanted into the injured region form and integrate into functional neuronal networks. Our recordings were conducted on cells in the injured region, also supporting the idea that similar cells could be used as potential treatment for lesions involving the neocortex.

In summary, we find that NPCs obtained from different sources of the embryonic brain (VZ/SVZ and GE) retain their identity, migrate to appropriate regions within the neocortex and integrate into the host tissue. Our results highlight the feasibility of this transplantation approach in treating cell loss following brain injury. Future studies using NPCs derived from more fundamental sources, such as induced pluripotent stem cells, will enhance the translational potential of our findings as the field moves forward in stem cell based therapies for neurodegenerative disorders.

## Author Contributions

KN participated in the study design, carried out all the experiments (tissue section, cell suspension and transplantation), immunoassays, data collection and analysis, and drafted the manuscript. NC did all the electrophysiology work and analysis, participated in manuscript drafting. MS performed transfections. SJ conceived the study, participated in its design, coordinated, and helped in writing the manuscript. All authors read and approved the final manuscript.

## Conflict of Interest Statement

The authors declare that the research was conducted in the absence of any commercial or financial relationships that could be construed as a potential conflict of interest.

## Funding

This work was conducted with funds provided by the CDMRP [PT074620 (SJ)].

## Abbreviations

aCSF, artificial cerebrospinal fluid; CMV, cytomegalovirus; E16, embryonic day 16; GE, ganglionic eminence; GFP, green fluorescent protein; MEM, minimum essential media; NPCs, neural progenitor cells; P0–P3, postnatal day 0–3; SVZ, subventricular zone; TBI, traumatic brain injury; VZ, ventricular zone.
